# Handmade Biopsy for Genotyping of In Vitro‐Produced Bovine Embryos

**DOI:** 10.1111/ahe.70128

**Published:** 2026-05-12

**Authors:** Luiz Sergio Almeida Camargo, Carolina Capobiango Romano Quintão, Julia Medeiros Cruz Lima de Martins, Marcos Vinicius Gualberto Barbosa Silva, Agostinho Jorge Reis dos Camargo, Celio Freitas, Daniele Ribeiro Lima de Reis, Marco Antonio Machado, Clara Slade Oliveira

**Affiliations:** ^1^ Embrapa Dairy Cattle Juiz de Fora Minas Gerais Brazil; ^2^ Fluminense Federal University Niterói Rio de Janeiro Brazil; ^3^ Agriculture Research Company of the Rio de Janeiro State (PESAGRO‐RIO) Niterói Rio de Janeiro Brazil

**Keywords:** biopsy, blastocyst, calf delivery, genomic selection, in vitro fertilization, pregnancy

## Abstract

Blastocyst biopsy allows genomic selection of pre‐implantation bovine embryos, reducing generation interval and enhancing the genetic gain. We aimed to evaluate the suitability of a simple biopsy procedure, performed by hands, using a splitting microblade and a stereoscope, on in vitro‐produced embryos. Crossbred *Bos taurus* x *B. indicus* blastocysts at Day 6 or 7 after in vitro fertilization (IVF) were biopsied and cultured in vitro for 48 h. Biopsy reduced (*p* < 0.01) embryo development as noted by the lower blastocoel formation/re‐expansion rates in both Day 6 (33.3% vs. 71.4%) and Day 7 (66.6% vs. 89.8%) blastocysts, when compared to non‐biopsied embryos. Biopsy on Day 7 decreased (*p* < 0.01) cell number (112.8 ± 6.3 vs. 149.9 ± 5.6) and increased (*p* < 0.01) apoptotic index (14.9 ± 1.4 vs. 6.2 ± 1.2). In a second experiment, the IVF blastocysts were biopsied in the farm, and cultured for 3 h. Re‐expanded blastocysts, corresponding to 78.3% biopsied embryos, were transferred to synchronized recipients. Pregnancy (57.4% vs. 62%), delivery (51.0% vs. 48%), gestation length (285.5 ± 2.0 vs. 284.6 ± 1.2 days) and birthweight (30.5 ± 1.8 vs. 29.9 ± 1.2 kg) were similar (*p* > 0.05) between handmade biopsy and non‐biopsied embryos. Whole genome amplification of biopsied samples resulted in 256.0 ± 155.9 ng/μL DNA. The mean call rate was 75.3% ± 4.3% and the imputed GEBV for 305‐day milk yield was 839.21 ± 61.6 kg. In conclusion, handmade biopsy can be performed at small laboratories and at farm level, contributing to reduce costs for embryo genomic selection. Pregnancy, calf delivery and birthweight were not compromised, and samples are suitable for downstream applications such as genotyping.

## 1 Introduction

Genomic selection has been an important tool for cattle breeding, with a high impact on the beef and dairy industry (Meuwissen et al. 2001; Wiggans et al. 2017). In US Holstein cattle, the genetic gain per year has increased drastically for milk, fat, and protein yields since the implementation of genomic selection (García‐Ruiz et al. 2016). Currently, several other developed and developing countries have implemented genomic selection in their breeding programs. The success of genomic selection depends on the reference population, where the effects of single nucleotide polymorphisms (SNP) are estimated based on the genotype and phenotype. The data is then used to predict the genomic estimated breeding value (GEBV) of a genotyped individual who is not in the reference population. That approach has been used by artificial insemination (AI) companies to select bulls with superior GEBV. Initially, the cost of genotyping was a limiting factor for the implementation of genomic selection, so that only companies could afford it. Fortunately, genotyping costs have been dropping along the years, which has made it more affordable for farmers, who can now genotype their female calves to make decisions at the farm level based on GEBV.

The association of genomic selection with reproductive technology, as multiple ovulations and embryo transfer (MOET), created the opportunity to accelerate genetic improvement. The best embryos produced by means of superovulation and uterus flushing after AI are selected by genomic selection to produce the next generation (Oliveira, Silva, et al. 2023). However, the number of embryos produced by MOET has dropped worldwide whereas the number of embryos produced by in vitro means (in vitro embryo production: IVP) has increased in the last years, overcoming the number of MOET embryos (Viana 2022). Indeed, some AI companies are now using IVP embryos to produce the next generations of bulls and commercial IVP laboratories have been producing thousands of embryos per month at lower cost to meet farmers' demand for genetically superior animals (Mullaart and Wells 2018). The lower costs of IVP and of genotyping make the perfect combination for farmers to start using genomic selection before the calf delivery, saving recipients and minimizing the chances of having genetically inferior calves at birth, since embryos can be selected based on their GEBV.

To calculate the GEBV of embryos, it is required to perform a biopsy to remove enough cells for genotyping. One of the main concerns is embryo viability after biopsy procedure, but several reports have shown that in vivo‐derived and in vitro‐produced biopsied embryos can develop and result in pregnancy at similar rates to non‐biopsied ones (de Sousa et al. 2017; Lopes et al. 2001; Machaty et al. 1993; Oliveira et al. 2017). Those data are encouraging and indicate that commercial IVP laboratories can adopt the embryo biopsy to offer this new service for farmers. However, embryo biopsy requires expensive equipment, such as micromanipulators, in an appropriate laboratory, which restrains small commercial companies from using embryo biopsy and increases the biopsy costs, contributing to make embryo genotyping less affordable for farmers. One alternative is performing embryo biopsy manually under a stereoscope and without micromanipulators. Embryo splitting performed in in vitro‐produced sheep embryos without micromanipulators resulted in a lower pregnancy rate and a higher rate of foetal loss (Morton et al. 2006). In bovine, although in vitro‐produced embryos split manually presented a reasonable in vitro developmental capability, the length of most elongated embryos on Day 17 was less than 100 mm, in contrast to non‐split embryos. In addition, the expression of pluripotency and trophoblastic markers was downregulated in those embryos (Velasquez et al. 2016). Embryo biopsy performed without micromanipulators in gene‐edited bovine blastocysts resulted in three (23%) pregnancies from 13 cryopreserved split embryos, although one of them died soon after birth (Wei et al. 2018). We reported previously no effect of embryo biopsy on pregnancy loss, mainly in the first trimester of gestation (Oliveira et al. 2017), but in that study there was no distinction between embryos biopsied with a splitting blade operated manually or with a micromanipulator.

Given the lack of consistent results for the use of embryo biopsy performed without micromanipulators and the importance that it can have for the application of embryo genomic selection programs, we investigated the effect of the day of biopsy on in vitro embryo development and quality, and the pregnancy and birth rates and birthweight of calves derived from in vitro‐produced blastocysts biopsied by handmade procedure. We also performed a preliminary investigation, with a small sample size, on whether the biopsies could provide sufficient genetic material, after performing whole genome amplification (WGA), for genotyping and estimation of the genomic breeding value (GEBV) for milk production of the biopsied blastocysts.

## 2 Material and Methods

All chemicals were from Sigma Chemical (St. Louis, MO, USA) unless stated otherwise. Procedures followed ethical guidelines for animal experimentation, and they were approved by the local Committee (CEUA–EGL, protocol 29/2013).

### 2.1 Experimental Design

The study was split into two experiments. The first experiment was carried out in the laboratory at Embrapa Dairy Cattle, with oocytes aspirated from ovaries collected at a commercial slaughterhouse from crossbred *Bos indicus* x *B. taurus* (Girolando cows with undefined genetic composition). The second experiment was carried out at Santa Monica Experimental Farm Station, with oocytes collected by ovum‐pick up (OPU) from *B. indicus* (Gir breed) cows and embryos produced in vitro in the laboratory located at the farm and according to its breeding program. Oocytes were in vitro matured and fertilized, and the presumptive zygotes were cultured in vitro until the blastocyst stage when a handmade biopsy was performed. In experiment 1 (four replicates), early blastocysts (*n* = 38) were biopsied on Day 6 (Day 0: in vitro fertilization) and cultured for 48 h. Blastocysts and expanded blastocysts (*n* = 78) were biopsied on Day 7 and cultured for 24 h. In both cases, the in vitro culture was performed to assess blastocoel formation/re‐expansion assessment. Blastocysts and expanded blastocysts biopsied on Day 7 were fixed after 24 h of in vitro culture to determine total cell number and apoptosis index. The control group was composed of non‐biopsied blastocysts. In experiment 2 (three replicates), blastocysts were biopsied on Day 7 after IVF (*n* = 74) and cultured for 3 h to allow blastocoel re‐expansion. Afterwards, re‐expanded blastocysts (*n* = 47) were transferred to synchronized recipients according to the number of available recipients (one embryo per recipient) and the pregnancy and delivery rate were determined. The control group was composed of non‐biopsied blastocysts. DNA extracted from biopsy samples of a few blastocysts (*n* = 8) was amplified by WGA and genotyped to investigate whether the provided genetic material would be sufficient to calculate the GEBV for milk production.

### 2.2 Ovaries Collection

Ovaries of *B. indicus* x *B. taurus* crossbred cows were collected at a commercial slaughterhouse (Fripai, Juiz de Fora, MG, Brazil) and transported to the laboratory in saline solution (0.9% NaCl with 0.1 g/L streptomycin) at 34°C–36°C within 3 h. Follicles with 2–8 mm diameter were aspirated and follicular fluid transferred to TALP HEPES medium. Cumulus cells‐oocytes complexes (COCs) searching was performed with a stereoscope and those with more than three compact layers of cumulus cells and homogeneous cytoplasm were selected for in vitro maturation.

### 2.3 Ovum Pick‐Up

Oocytes were obtained from Gir ( *B. indicus* ) cows by ultrasound‐guided transvaginal follicular aspiration performed with disposable 18‐gauge needles and a vacuum pressure of 90 mmHg using a transvaginal 7.5 MHz convex transducer coupled to an ultrasound equipment (Mindray DP2200, Shenzhen, China). Follicular fluid obtained from 2 to 8 mm diameter follicles was collected in 50 mL tubes containing Dulbeco's PBS supplemented with 50 IU heparin and 1% fetal calf serum (FCS) solution, at 37°C. Afterwards, follicular fluid was filtered and COCs searching performed with stereoscope. COCs with at least one compact layer of cumulus cells and oocyte with homogeneous cytoplasm were selected for in vitro maturation.

### 2.4 In Vitro Maturation, Fertilization an Embryo Culture

In the experiment 1, in vitro maturation of COCs was performed for 24 h in TCM‐199 (Gibco Life Technologies Inc., Grand Island, NY, USA) supplemented with 10% estrous cow serum, 20 μg/mL follicle stimulating hormone (FSH; Pluset, Calier, Spain) and 0.05 mg/50 IU per mL of streptomycin/penicillin in a humidified atmosphere of 5% CO_2_ in air and 100% humidity. IVF was performed with Holstein frozen–thawed semen. Semen was centrifuged at 3600 *g* for 7 min in Percoll discontinuous density gradient (45% and 90%). The pellet was resuspended in Fert‐Talp medium and centrifuged again at 520 *g* for 5 min. Spermatozoa/oocytes co‐incubation was performed between 20 and 21 h in Fert‐TALP supplemented with 20 μg/mL of heparin and 6 mg/mL of fatty acid free BSA fraction V, covered with mineral oil, in a humidified atmosphere of 5% CO_2_ and 38.5°C in air. After IVF, the presumptive zygotes were completely denuded by vortexing them in 0.1% hyaluronidase solution and then cultured in a modified CR2aa medium with 2.5% of fetal calf serum (FCS) under 5% CO_2_, 5% O_2_ and 90% N_2_ with saturated humidity at 38.5°C.

In the experiment 2, COCs were in vitro maturated for 24 h in TCM‐199 medium supplemented with 10% FCS, 1.0 μg/mL FSH (Folltropin, Bioniche, Belleville, Canada), 50 μg/mL hCG (Profasi, Serono, Sao Paulo, Brazil), 1.0 μg/mL estradiol and 83.4 μg/mL amikacin. Afterwards, oocytes were in vitro‐fertilized with Holstein or Gir X‐sorted semen for 16–18 h using similar procedure described in experiment 1. After IVF, presumptive zygotes were partially denuded by vigorous pipetting and cultured in SOF medium supplemented with 2.5% FCS and 6 mg/mL BSA under 5% CO_2_ in air with saturated humidity at 38.5°C.

### 2.5 Embryo Biopsy

**FIGURE 1:**
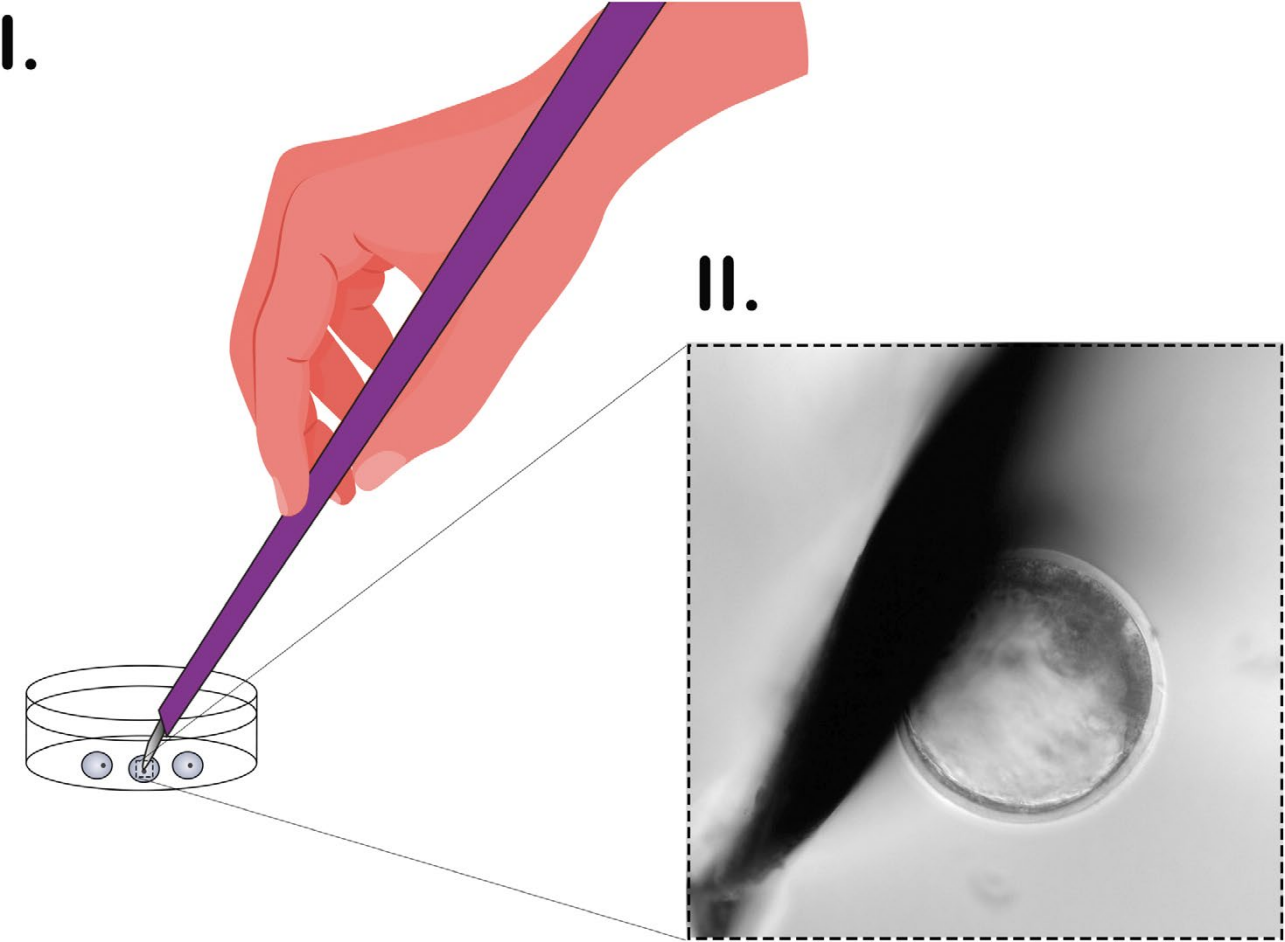
Handmade biopsy procedure. Microblade is positioned by hands inside a 20 μL buffered medium drops covered by mineral oil containing one blastocyst. The microblade splits the embryo, separating the biopsy sample from the trophoectoderm portion of the blastocyst. (I) Schematic representation of the procedure. (II) Microscopic view of the microblade and the blastocyst just before biopsy sample separation.

**FIGURE 2:**
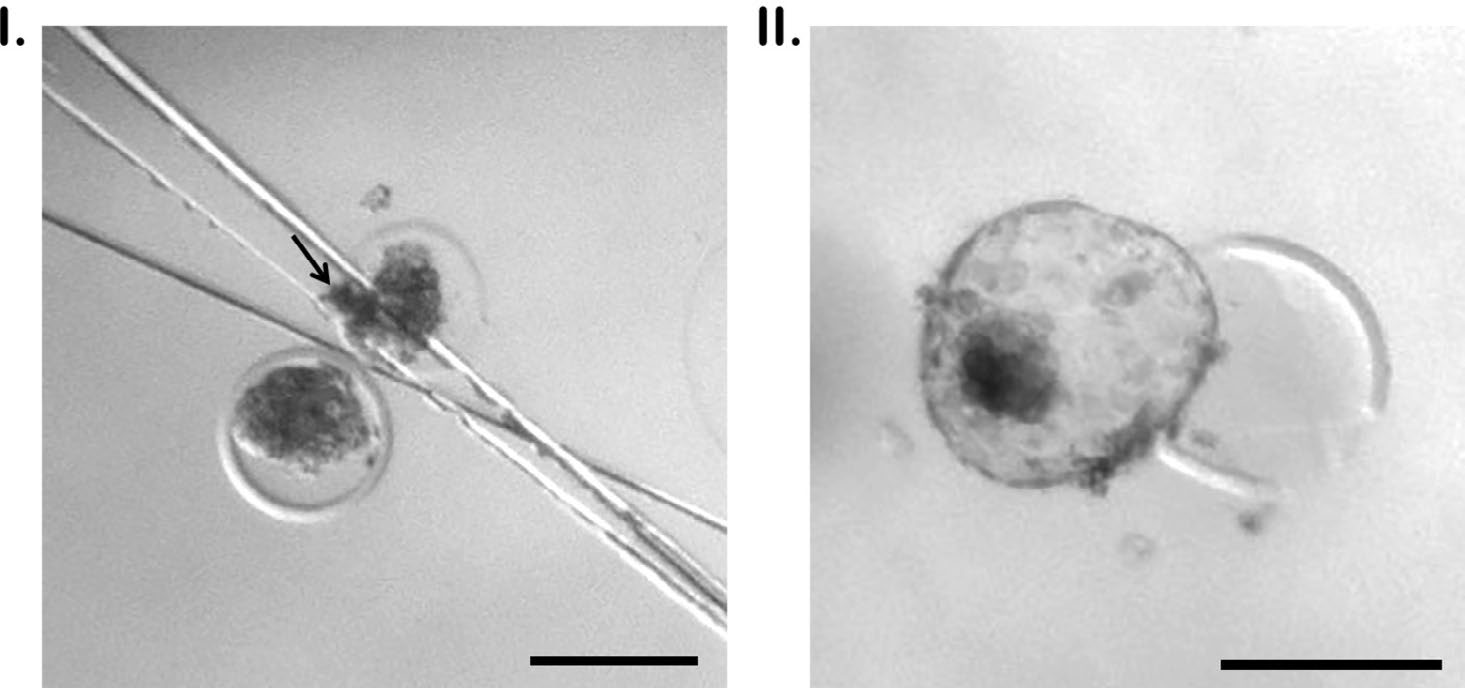
Embryo morphology following biopsy procedure. (I) A blastocyst was subjected to handmade biopsy, and this image shows its morphology just after the procedure. Arrow indicates biopsy sample. (II) Blastocyst morphology 48 h after handmade biopsy.

Blastocysts exhibiting no extruded cells or debris were washed twice in buffered medium (HEPES‐SOF or TALP HEPES) without serum and then transferred individually to 20 μL drop covered by mineral oil. Embryo was positioned in the middle of the drop under a stereoscope (30 and 40× magnification) and a splitting microblade (Ultra Sharp Splitting blade, Bioniche, Canada, Angled at approximately 15°, with a blade length of 2 mm, a maximum thickness of 100 μm, and an ultrasharp tip) was operated manually (Figure 1) to split the blastocoel and remove between 15% and 25% of total cell mass. When required, a small scratch was made using the microblade to stick the embryo to the bottom of the dish (Figure 2I). Afterwards, the embryos were recovered, washed, and cultured individually.

The handmade biopsy procedure was performed with strict adherence to the points described below. First, the operator was an experienced embryologist who underwent at least 5 days of training, performing biopsies on approximately 20 embryos per session. Second, the use of a serum‐free medium is highly recommended, as it facilitates embryo manipulation. Third, embryo stabilization is improved when a scratch is present on the bottom of the dish; the embryo is positioned within this area, and the blade is placed at the centre of the embryo to gently apply downward pressure. Just before the incision (when the embryo is slightly compressed), the blade is directed laterally to excise approximately 20% of the trophectoderm. Fourth, extreme care must be taken to detach the biopsy from either the blade or the bottom of the dish, which can be achieved by gently pipetting medium until it is released or by carefully nudging it with the pipette tip to form a compact cluster of cells. Finally, special attention is required to ensure that the biopsy sample is properly transferred into the tube prior to freezing.

In the experiment 1, blastocysts were cultured in CR2aa medium supplemented with 2.5% FBS under 5% CO_2_, 5% O_2_ and 90% N_2_ at 38.5°C. Blastocysts biopsied on Day 6 were cultured for 48 h (until Day 8 after IVF) and blastocysts biopsied on Day 7 were cultured for 24 h (until Day 8 after IVF) in order to evaluate the in vitro development, assessed by blastocoel formation/re‐expansion (Figure 2II). In the experiment 2, biopsied Day 7 blastocysts were cultured for 3 h in HEPES‐SOF medium on a warming plate at 37°C to assess the blastocoel re‐expansion. Afterwards, re‐expanded embryos were loaded in 0.25 mL straws and transferred to synchronized recipients to assess pregnancy and delivery rate. Control groups consisted of non‐biopsied blastocysts in both experiments. Samples from biopsied blastocysts were frozen individually in a DNAase‐free cryotube containing 1 μL Dulbecco's PBS and stored in −20°C.

### 2.6 Total Cell Number and Apoptosis Assessment

Blastocysts on Day 8 after IVF were fixed in paraformaldehyde at 4°C and submitted to terminal deoxyribonucleotidyltransferase‐mediated dUTP‐digoxigenin nick end‐labelling (TUNEL) according to the manufacturer's instructions (Dead End Fluorimetric TUNEL System, Promega, Madison, USA). Briefly, fixed blastocysts were permeabilized with 0.2% Triton X‐100 (Promega) in Dulbecco's PBS. Positive control embryos were previously treated with DNase. After permeabilization, samples were incubated in 100 μL drops with reagent mix containing enzyme solution and 90% staining solution (dUTP fluorescein conjugate) for 1 h at 37°C in a dark humid chamber. Negative control samples were incubated only in the staining solution without enzyme solution. Samples were then stained with Vectashield (Vector Laboratories Inc., Burlingame, CA, USA) plus 4′6‐diamidino‐2‐phenylindole (DAPI) and mounted on slides for evaluation by fluorescence microscopy. Total cell number and apoptotic cell number per embryo were counted and the apoptotic cell index was calculated as the ratio of apoptotic cell/total cell number × 100.

### 2.7 DNA Extraction and Whole Genome Amplification (WGA)

DNA extraction and WGA were performed by multiple displacement amplification (MDA) technique using Single Cell GenomiPhi DNA Amplification kit (GE Healthcare, Buckinghamshire, UK) according to the manufacturer's instructions. Detailed protocol is described in Oliveira et al. (2026). Briefly, cells were lysed by mixing 1 μL sample with 1 μL lysis buffer and incubated at 65°C for 10 min. Reaction was stopped by adding 1 μL of neutralizing buffer. Master mix solution containing enzyme, reaction buffer and water was added to the extracted DNA to obtain a final volume of 20 μL per reaction and then incubated at 30°C for 2 h. Reaction was inactivated by incubating it at 65°C for 10 min. Amplified DNA was quantified by 2100 Bioanalyzer (Agilent Technologies, Santa Clara, USA), according to the manufacturer's instructions.

### 2.8 Genotyping, Imputation and Estimation of Genomic Breeding Value

Genotyping of biopsy samples was performed by Neogen do Brasil (Pindamonhangaba, Brazil) using the Bovine SNP50k chip (Illumina Inc., San Diego, USA). The phenotypic data consisted of 305‐day milk yield (305MY) records from Gir animals obtained from herds supervised by the Brazilian Association of Zebu Breeders (ABCZ), Brazilian Association of Gir Dairy Cattle Breeders (ABCGIL), and Brazilian Association of Girolando Breeders Associations. Those animals were genotyped with different assays: Illumina Bovine HD Bead Chip (HD‐777 K), BovineSNP50 Bead Chip v2 (50K), GGP Indicus (34 K), Z‐Chip (30 K), and GGP Indicus (50 K). For the imputation process, the reference population included only animals genotyped with the HD‐777 K assay.

The calculation of the imputed GEBV was performed at Bioinformatics and Animal Genomics Laboratory (Embrapa Dairy Cattle, Juiz de Fora, MG), which is responsible for the annual genetic and genomic evaluations of Gir dairy cattle in Brazil. Genomic breeding values were estimated using single‐step genomic BLUP performed with the BLUPF90 software family (Misztal et al. 2002), as described (Oliveira, Silva, et al. 2023). The accuracy (ACC) of the GEBV was estimated for each embryo biopsy/animal and for each scenario as follows:

$${\mathrm{ACC}}_{i}=\sqrt{1-\left(\frac{{\mathrm{SD}}_{i}^{2}}{\sigma_{u}^{2}}\right)}$$

in which ${\mathrm{ACC}}_{i}$ is the accuracy of breeding value for animal *I* and SD is the estimates standard deviation from GBLUP equations.

### 2.9 Estrous Synchronization and Embryo Transfer

Estrous of crossbred recipients was synchronized by a progesterone/oestrogen‐based protocol. Hormones were purchased from Ourofino (Cravinhos, Brazil). Recipients received a vaginal device containing 1 g progesterone and 2 mg estradiol benzoate. Seven days later, recipients received one cloprostenol injection (0.5 mg), and the progesterone device was removed. On the following day, recipients were treated with 1 mg estradiol benzoate. Blastocysts were randomly transferred to recipients with corpus luteum on the 9th day after progesterone device removal. Pregnancy was assessed by ultrasound evaluation at the 30th and 90th day after embryo transfer. Birthweight was recorded at calf's delivery.

### 2.10 Statistical Analysis

Data of blastocoel formation/re‐expansion, pregnancy and birth rates between biopsy and control groups were compared by chi‐square. Total cell number, apoptosis cell number, apoptosis index and birthweight were compared by analysis of variance using General Linear Model procedure of SAS package (version 9.1, SAS, Cary, USA). Values are shown as mean ± standard error of means (S.E.M.), except for GEBV, which values are shown as mean ± standard deviation. Differences were considered significant at the 95% confidence level (*p* < 0.05).

## 3 Results

Experiment 1 evaluated the in vitro ability of blastocysts to develop after biopsy performed on Day 6 or 7 after IVF. Blastocoel formation/re‐expansion after biopsy was used as a measure of development. Almost half of blastocysts on Day 6 was at early stage (33/67; 49.25%) in contrast to Day 7, when blastocysts were mainly at expanded stage (45.85%, 72/157) and only 11.41% (18/157) were at early stage. We found that biopsy performed on Day 6 and 7 after IVF impaired (*p* < 0.01) blastocoel formation/re‐expansion on Day 8 (Table 1). The ratio of blastocoel formation/re‐expansion rate between biopsied and control embryos was 0.46 for biopsies performed on Day 6 and 0.74 for Day 7. Then, we investigated whether the number of total cells and apoptosis index could be affected by the biopsy performed on Day 7, once blastocysts on Day 7 are the best choice for embryo transfer (Hasler 1998) and also because of the high ratio of blastocoel formation/re‐expansion rate between biopsied and control embryos. Biopsy decreased the total cell number and increased the number of apoptotic cells (*p* < 0.01). Biopsied blastocysts had 24.7% lesser cells than non‐biopsied blastocyst, resulting in higher apoptotic index than control (Table 2).

**TABLE 1:** Blastocoel formation/re‐expansion on day seven and day eight post in vitro fertilization (IVF) of blastocysts biopsied day six or seven post IVF.

Groups	*N*	Blastocoel formation/re‐expansion on Day 7	Blastocoel formation/re‐expansion on Day 8
Biopsy on Day 6			
Biopsied embryos	39	20 (51.3%)^b^	13 (34.2%)^a^
Control embryos	28	21 (75.0%)^a^	20 (71.4%)^b^
Biopsy on Day 7			
Biopsied embryos	78	—	52 (66.6%)^a^
Control embryos	79	—	71 (89.8%)^b^

*Note:* Blastocysts biopsied on Day 6 were cultured for 48 h and blastocysts biopsied on Day 7 were cultured for 24 h. ^a,b^Superscript letters in the same column within the same biopsy day indicate differences (*p* < 0.05) between control and biopsied embryos. Data from four replicates. Chi‐square test.

**TABLE 2:** Total cell number and apoptosis of re‐expanded biopsied blastocysts.

Groups	*N*	Total Cell number	Apoptosis cell number	Apoptotic index
Biopsied embryos	20	112.8 ± 6.3^b^	16.7 ± 1.8^a^	14.9 ± 1.4^a^
Control embryos	18	149.9 ± 5.6^a^	9.6 ± 1.9^b^	6.2 ± 1.2^b^

*Note:* Blastocyst biopsied on day 7 post IVF were cultured for 24 h. Values are shown as mean ± SEM. ^a,b^Superscript letters indicate significant differences (*p* < 0.01) between control and biopsied embryos. Data from four replicates. Analysis of variance.

Experiment 2 evaluated the effects of handmade embryo biopsy on pregnancy, calf delivery, and birthweight. Blastocysts were biopsied on Day 7 after IVF and cultured for 3 h in buffered solution on a warming plate before being transferred to synchronized recipients. The proportion of biopsied blastocysts displaying a re‐expanded blastocoel within 3 h after biopsy was 78.3% (58/74 blastocysts). Forty‐seven re‐expanded biopsied and 50 control blastocysts were transferred to recipients. There was no difference (*p* > 0.05) in pregnancy rate at the 30th and 90th day after embryo transfer and in birth rate between biopsied and control blastocysts (Table 3). There was no difference (*p* > 0.05) in gestation length and birthweight between calves derived from biopsied (285.5 ± 2.0 days and 30.5 ± 1.8 kg, *n* = 24) or control (284.6 ± 1.2 days and 29.9 ± 1.2 kg; *n* = 24) embryos.

**TABLE 3:** Pregnancy and birth rate of gestations derived from blastocysts biopsied on Day 7 after in vitro fertilization.

Group	*N*	Pregnancy at 30th day	Pregnancy at 90th day	Birth rate
Biopsied embryos	47	27 (57.4%)	27 (57.4%)	24 (51.0%)
Control embryos	50	31 (62%)	27 (54.0%)	24 (48.0%)

*Note:* Biopsied blastocysts were cultured for 3 h and those with re‐expanded blastocoel were transferred to synchronized recipients. No significant difference between groups (*p* > 0.05). Data from three replicates. Chi‐square test.

Genetic material of biopsies obtained from eight blastocysts was amplified by WGA and genotyped with SNP50k chip to calculate the imputed GEBV for 305‐day milk yield. DNA concentration after WGA was 256.0 ± 155.9 ng/μL in a final volume of 20 μL. DNA fragment length ranged between 2.3 ± 0.59 and 5.5 ± 1 kb. The mean call rate of biopsy samples was 75.3% ± 4.3%, with 62.5% (5/8) of samples with call rate higher than 80%. The imputed GEBV for 305‐day milk yield was 839.21 ± 61.6 kg with an accuracy of 50.6% ± 3.7%.

## 4 Discussion

In this study, we evaluated the effects of a handmade embryo biopsy procedure performed without micromanipulators, a few hours before transferring IVP embryos to recipients. Despite reducing the total cell number and increasing the proportion of apoptotic cells in blastocysts, handmade biopsy does not have any significant effect on pregnancy, gestation length, and birth rate or even on birthweight. Whole genome amplification of biopsy samples resulted in DNA concentrations that are suitable for downstream applications as SNPs genotyping. With this approach, embryos can be biopsied in small commercial laboratories or on the farms without requiring expensive equipment and a well‐established facility.

In vitro evaluations found that handmade embryo biopsy performed on Days 6 or 7 after IVF restricted in vitro development, assessed by the ability of blastocysts to re‐expand the blastocoel during in vitro culture. One problem perceived on Day 6 was to perform biopsy in early blastocysts. Almost half of blastocysts (49.25%) were at early stage, and it was difficult to distinguish the inner cell mass (ICM) from blastocoel before sectioning the embryos, resulting in a more challenging and time‐consuming biopsy procedure. On the other hand, embryos on Day 7 after IVF were at blastocyst or expanded blastocyst stages, which made it easy to locate and split the blastocoel and may have contributed to a high ratio of blastocoel re‐expansion rate between biopsied and control embryos.

Total cell number and apoptosis were evaluated in biopsied blastocysts cultured in vitro for 24 h. Biopsied blastocysts had almost 25% lesser cells than non‐biopsied blastocysts, which was within the proportion of cells intended to be removed by the biopsy procedure (between 15% and 25%). A previous study with bovine embryos biopsied at the 8–16 cell stage reported no difference in total cell number after reaching blastocyst stages (Polisseni et al. 2010). In the present study, the lower number of total cells was expected, as biopsied embryos were cultured for only 24 h before total cell counting.

Number of apoptotic cells and apoptotic index increased in biopsied blastocysts. Apoptosis is a cell death process required to eliminate damaged cells and can be triggered by stressful factors (Fulda et al. 2010). In bovine blastocysts, the occurrence of apoptosis is higher in the ICM than in trophoblast (Oliveira et al. 2010). The handmade procedure may have caused some injuries to the embryo and damaged some cells, what could trigger the apoptosis cascade and increase the number of apoptotic cells. In addition, the biopsy procedure was performed to remove trophoblast from the embryo, whose cells have a lower incidence of apoptosis than ICM cells. Thus, the higher apoptotic cell index in biopsied blastocysts is likely a combination of a lower number of total cells, a higher number of apoptotic cells, and the removal of non‐apoptotic cells in the trophectoderm by biopsy procedure.

In the second experiment, the IVP was carried out in an experimental farm (Santa Monica, Embrapa Dairy Cattle), following the requirements of the farm's breeding program, using oocytes from selected donors' cows, sex‐sorted sperm from different bulls and commercial media, and using the farm facility. Although the conditions were different from the first experiment, which was carried out in a research facility under a better controlled environment, this second experiment allowed us to demonstrate the feasibility of the handmade biopsy under circumstances found in the farms. Due to the re‐expansion rates found in the first experiment, we chose to perform embryo biopsy only in blastocysts on Day 7 post IVF. Three hours of culture after biopsy was sufficient to allow most of the embryos to re‐expand the blastocoel (78.3%), totally or partially. The handmade biopsy had no negative impact on pregnancy, gestation length and birth rates and on birthweight. It was already shown that embryo biopsy using micromanipulators has no effect on pregnancy rates of bovine IVP blastocysts (de Sousa et al. 2017; Oliveira, Camargo, et al. 2023; Oliveira et al. 2017) and birthweight (Oliveira, Camargo, et al. 2023; Oliveira et al. 2017). A previous study reported that embryos biopsy performed by microblade increased the damage to zona pellucida and affected the pregnancy rate when compared to needle biopsies with micromanipulators (Cenariu et al. 2012). In this present study we found that biopsied embryos lost the zona pellucida; however, there was no effect on pregnancy establishment, showing that the handmade biopsy procedure can be used safely in bovine blastocysts.

Interestingly, in the first experiment we found that the handmade biopsy can increase the apoptotic index in the biopsied blastocysts, what can suggest a negative impact on embryo viability when compared to control embryos. However, in the second experiment, no effects of handmade biopsy on pregnancy and birth rate were detected. Although the conditions of in vitro embryo production present some differences between experiments, which may influence the apoptosis rate, our data suggest that the proportion of apoptotic cells in biopsied blastocysts may not be enough to impair the embryo's ability to establish a gestation. A hypothesis is that a better environment can be provided by the endometrium than in vitro culture, enhancing embryo recovery after biopsy. Indeed, it was shown that the exposure of Day 7‐IVP blastocysts to the uterine environment can improve post‐hatching development (Machado et al. 2013).

One problem with samples from biopsied embryos is the small number of cells resulting in low DNA input for genotyping, which requires a WGA. Previous study reported that 30 cells was the minimum number of cells from biopsied bovine embryo required for WGA performed by multiple displacement amplification (MDA) technique, and that 10 cells resulted in genomic coverage of 72.4%, with only 31.25% of samples showing genotyping success better than 85% (Lauri et al. 2013). Other study reported that high DNA input (10 ng) resulted in high call rate (> 90%) using the MDA technique offered by two different WGA kits, but low DNA input (15 cells) resulted in average call rate below 70% and 90%, differing accordingly to the kit used for WGA (Saadi et al. 2014). In our study we used the MDA technique to amplify the whole genome of eight biopsy samples. Although the DNA concentration after WGA was 256.0 ng/μL, it ranged from 14.1 to 1030.8 ng/μL. The call rate was 75.3% in average, ranging from 54% to 87.6%, with five out eight samples above 80%. Lower call rates are expected for amplified DNA samples, as whole‐genome amplification (WGA) may not occur uniformly across the genome. In our experience, no association was observed between DNA yield and call rate, suggesting that even lower DNA yields can result in high genomic coverage (Oliveira, book chapter). This is consistent with the fact that the assay used for multiple displacement amplification (MDA) is designed for single‐cell analysis. Therefore, the primary concern is DNA fragmentation prior to MDA, which should be minimized through rapid freezing and appropriate sample storage.

One limitation of the handmade biopsy approach is the lack of precision in obtaining a consistent number of cells from each embryo. Although biopsies were performed with the aim of removing approximately 15%–25% of the total cell mass—estimated at around 18–30 cells, assuming a typical Day‐7 IVP blastocyst contains ~120 cells—the actual number of cells collected per sample may vary. This variability can influence the efficiency of whole‐genome amplification (WGA), leading to substantial differences in DNA concentration. While the exact number of cells in each biopsy cannot be determined due to the downstream use of WGA, such variation may impact amplification efficiency and, consequently, the call rate in some samples. Larger blastocysts, with a higher total cell count, may therefore be more suitable for handmade biopsy procedures.

The call rate cut‐off to run further genomic analysis can range from 80% to 95% worldwide (Cooper et al. 2013). Nevertheless, genotype imputation can be employed to improve the data quality in samples with low call rate (Oliveira, Camargo, et al. 2023). Missing genotypes can be imputed based on parental genotypes (Boichard et al. 2012). Imputation can correct over 95% of errors found in genotyped samples from biopsied embryos (Saadi et al. 2014). In Gir breed, imputation of young bulls has shown to be highly accurate due the relationship between imputed samples and reference population (Boison et al. 2017). In the present study, the GEBVs for 305‐day milk yield of eight embryos were estimated after imputation using the same strategy reported by Oliveira, Silva, et al. (2023) and demonstrated the suitability of genetic material collected by handmade biopsy for calculation of GEBV.

In conclusion, handmade embryo biopsy can be performed on in vitro‐produced blastocysts on Day 7 without compromising pregnancy, calf delivery, and birthweight. Biopsy samples can be suitable for downstream applications after whole genome amplification, as genotyping. This approach is easy to perform and does not require specific and expensive equipment. It can be performed in small and simple commercial laboratories and on‐farm, and it can contribute to make the embryo genomic selection more affordable for farmers. Further studies are required to evaluate the cryosurvival of those handmade biopsied IVP blastocysts.

## Author Contributions

Luiz Sergio Almeida Camargo and Clara Slade Oliveira: conceptualization, supervision, methodology, original draft, investigation, review and editing. Marcos Vinicius Gualberto Barbosa Silva: investigation, formal analysis, review and editing. Carolina Capobiango Romano Quintão, Julia Medeiros Cruz Lima de Martins, Celio Freitas, Agostinho Jorge Reis dos Camargo, and Daniele Ribeiro Lima de Reis: investigation, validation, review and editing. Marco Antonio Machado: resources, review and editing.

## Conflicts of Interest

The authors declare no conflicts of interest.

## Data Availability

The data that support the findings of this study are available from the corresponding author upon reasonable request.
